# Hexagonal Boron Nitride–Enhanced Optically Transparent Polymer Dielectric Inks for Printable Electronics

**DOI:** 10.1002/adfm.202002339

**Published:** 2020-06-02

**Authors:** Xiaoxi Zhu, Leonard W. T. Ng, Guohua Hu, Tien‐Chun Wu, Doo‐Seung Um, Nasiruddin Macadam, Tawfique Hasan

**Affiliations:** ^1^ Cambridge Graphene Centre University of Cambridge Cambridge CB3 0FA UK; ^2^ Department of Electronic Engineering The Chinese University of Hong Kong Shatin Hong Kong S. A. R.

**Keywords:** 2D materials, dielectrics, functional inks, hexagonal boron nitride, K‐bar coatings, printed electronics

## Abstract

Solution‐processable thin‐film dielectrics represent an important material family for large‐area, fully‐printed electronics. Yet, in recent years, it has seen only limited development, and has mostly remained confined to pure polymers. Although it is possible to achieve excellent printability, these polymers have low (≈2–5) dielectric constants (ε_*r*_). There have been recent attempts to use solution‐processed 2D hexagonal boron nitride (*h*‐BN) as an alternative. However, the deposited *h*‐BN flakes create porous thin‐films, compromising their mechanical integrity, substrate adhesion, and susceptibility to moisture. These challenges are addressed by developing a “one‐pot” formulation of polyurethane (PU)‐based inks with *h*‐BN nano‐fillers. The approach enables coating of pinhole‐free, flexible PU+*h*‐BN dielectric thin‐films. The *h*‐BN dispersion concentration is optimized with respect to exfoliation yield, optical transparency, and thin‐film uniformity. A maximum ε_*r*_ ≈ 7.57 is achieved, a two‐fold increase over pure PU, with only 0.7 vol% *h*‐BN in the dielectric thin‐film. A high optical transparency of ≈78.0% (≈0.65% variation) is measured across a 25 cm^2^ area for a 10 μm thick dielectric. The dielectric property of the composite is also consistent, with a measured areal capacitance variation of <8% across 64 printed capacitors. The formulation represents an optically transparent, flexible thin‐film, with enhanced dielectric constant for printed electronics.

## Graphical abstract

A hexagonal boron nitride‐enhanced, flexible, and optically transparent thin‐film dielectric is reported. The pin‐hole‐free composite is fabricated by K‐bar coating and shows up to a two‐fold increase in dielectric constant. The optical transparency is consistent, with 0.65% variation. The formulation is suitable for large‐area applications such as printed capacitors and transparent capacitive touch surfaces.

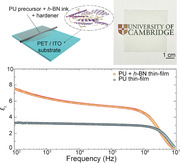

## 1 Introduction

Printed electronics hold great potential for flexible, wearable, and large form‐factor devices.^[^ ^1^, ^2^, ^3^ ^]^ The most basic materials platform for this requires conductive, semiconductive, and dielectric components. Although not widely researched compared to their counterparts, dielectric materials play a pivotal role in the functioning of many fully printed devices.^[^ ^4^, ^5^ ^]^ For instance, as passive components, capacitive structures are necessary for most printed electronic systems, forming the basis of a wide variety of circuits, including resonators, filters, memory elements, and capacitive strain/touch sensors. In addition to the printability, these applications demand dielectric materials that not only offer but also retain stable electrical properties and mechanical integrity over the device lifetime.

Traditionally, cross‐linked polymers such as Poly(4‐vinylphenol) (PVP) have been used as printable dielectrics^[^ ^6^, ^7^, ^8^ ^]^ as they show superior scalability, and adaptability into various device configurations compared to their ceramic counterparts. Although their rheology can be modified for large area printing and coating, these polymers have relatively low ε_*r*_ (≈2–5^[^ ^9^, ^10^, ^11^ ^]^) values. Among these, Polyurethane (PU) represents a class of polymers that are widely used in graphics, and functional printing and coatings industry^[^ ^12^ ^]^ due to its high tensile strength, scratch, corrosion, and solvent resistance.^[^ ^13^, ^14^ ^]^ These properties also make PU and related dielectric composites interesting for electronics, but with a moderate ε_*r*_ (≈3–4^[^ ^15^, ^16^, ^17^ ^]^). The limitations of these polymers have prompted the search for other readily printable, thin‐film dielectric materials.

2D hexagonal boron nitride (*h*‐BN), an insulating analogue of graphene, is frequently used as a dielectric screening layer for graphene and other 2D materials for device applications^[^ ^20^, ^21^, ^22^ ^]^ due to its wide bandgap (≈6 eV).^[^ ^23^, ^24^, ^26^ ^]^ This approach has also been applied to printable electronics, using solution‐phase exfoliation of *h*‐BN and its incorporation into functional inks.^[^ ^25^, ^27^, ^28^ ^]^ To this end, *h*‐BN has been exfoliated in water with dispersants^[^ ^18^, ^30^ ^]^ and in alcohols^[^ ^19^ ^]^ for the fabrication of capacitors and gate dielectrics by inkjet printing or spray coating. However, these approaches have thus far offered low/moderate dielectric properties with ε_*r*_ typically < 2–3, with a maximum reported value of ≈6.^[^ ^19^, ^22^ ^]^ In addition, the thin films fabricated from these exfoliated *h*‐BN dispersions form a porous structure after solvent evaporation. This could further compromise the already low breakdown voltage and mechanical integrity of the dielectrics due to environmental perturbations.

A potential solution to the above could be to incorporate *h*‐BN nanofillers directly into dielectric polymers. An attempt at this strategy with *h*‐BN enhanced, thermally cross‐linked divinyltetramethyldisiloxane‐bis(benzocyclobutene) (BCB) nanocomposite gives a stable dielectric constant (ε_*r*_ ≈ 3) at high temperature (250 °C) and frequency (10^6^ Hz).^[^ ^32^ ^]^ While the addition of 10 vol% *h*‐BN in the nanocomposite improves the heat dissipation, the dielectric does not demonstrate sufficiently high ε_*r*_, and cannot be readily used in printed electronics manufacturing.^[^ ^32^ ^]^

Here, we demonstrate that incorporation of only 0.7 vol% exfoliated *h*‐BN in the resultant PU+*h*‐BN thin‐film results in a two‐fold increase in its dielectric properties (ε_*r*_ ≈ 7.57) across 100 to 10 × 10^6^ Hz frequency range. We use a “one‐pot” process, exfoliating and stabilizing *h*‐BN flakes into a polyol (PU precursor) with 2‐Butoxyethanol (2‐BE) solvent. Following coating of the stable dispersion and room temperature cross‐linking, the flexible 10 µm thin film shows high optical transparency (≈78.0% at 550 nm), with a transmittance variation of only ≈0.65% across a 5 × 5 cm^2^ area. The dielectric property of the transparent thin film is also uniform, showing < 8% variation in ε_*r*_ among 64 fabricated capacitors. Our approach in enhancing the dielectric constant of a commonly used printable polymer, while keeping high optical transparency, could be of interest for large area, fully printed transparent and flexible capacitive structures, such as low pass filters and touch sensors.

## 2 Results and Discussion

**Figure 1:**
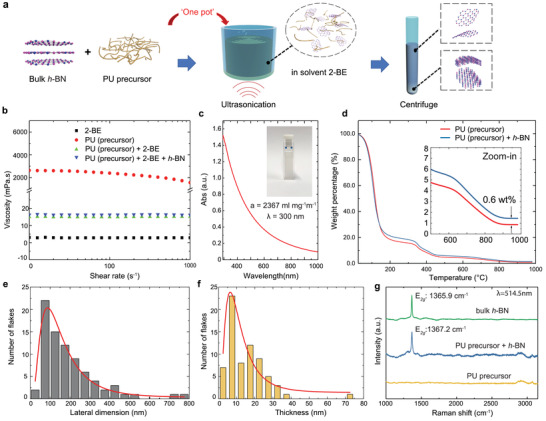
a) A schematic of the “one‐pot” processing for UALPE of *h*‐BN and ink formulation. b) Rheology measurements of 2‐BE solvent, PU (precursor), PU (precursor)+2‐BE and PU (precursor)+2‐BE+*h*‐BN. c) Optical absorption spectrum of exfoliated *h*‐BN in ink. Inset: Diluted sample in cuvette for measurement. d) TGA of the PU precursor with and without *h*‐BN. Inset: Close‐up of the high temperature region of the TGA measurements. AFM statistics indicating the e) lateral size and f) thickness of exfoliated *h*‐BN flakes. g) Raman spectra for bulk *h*‐BN, plain PU precursor, and PU precursor+*h*‐BN ink. The excitation wavelength is 514.5 nm.

Our dielectric ink formulation incorporates *h*‐BN into PU with the assistance of a solvent. This “one‐pot” process allows exfoliation and stabilisation of *h*‐BN flakes directly into the PU precursor/solvent system (**Figure** **1**a), as opposed to the commonly used two‐step process involving exfoliation in a solvent, followed by mixing with the host polymer. In our process, the liquid PU polymer precursor (polyol) simultaneously works as the dispersant for the ink, and after cross‐linking and solvent evaporation, as the binder for the dielectric coating. As shown in Figure 1b, the high viscosity of the pure PU precursor (≈1500 mPa.s at 1000 s^−1^ shear rate) is not conducive to create sufficient cavitation (the formation, growth, and eventual collapse of bubbles in liquid to generate high shear forces)^[^ ^34^ ^]^ for the exfoliation of 2D crystals.^[^ ^35^ ^]^ We therefore introduce 2‐BE as the solvent (viscosity 2.9 mPa.s, Sigma Aldrich) to reduce the overall viscosity to ≈16 mPa.s. As a solvent, 2‐BE is widely used in many domestic and industrial products, and is non‐toxic, cost‐effective, and environmentally friendly. It has a moderate boiling point (171 °C) compared to solvents conventionally used for exfoliation and stabilisation of 2D crystals (e.g., N‐Methyl‐2‐pyrrolidone NMP: ≈204 °C). The 16 mPa.s viscosity we choose cannot only support efficient liquid‐phase exfoliation of *h*‐BN flakes, but also stabilize the dispersions against re‐aggregation and sedimentation. We note that the required typical viscosity ranges are 4–30 mPa.s for inkjet printing, 100–1k mPa.s for gravure printing, 1k–2k mPa.s for flexographic printing, and 1k–10k mPa.s for screen printing which could enable high resolution patterning.^[^ ^33^ ^]^ The viscosity of our formulated ink can be tuned to make them more suitable for these techniques, for example, by adding more solvent (2‐BE, viscosity ≈2.9 mPa.s) or more binder (≈1500 mPa.s at 1000 s^−1^ shear rate). However, we note that “printability” and “functionality” are two different criteria, both of which need to be fulfilled for functional printing.^[^ ^33^ ^]^ Although the viscosity range of our formulation can be tuned for various printing technologies, a comprehensive rheology and ink design study should be carried out, with systematic investigation on the thin‐film uniformity (for example, against de‐wetting^[^ ^6^ ^]^ and coffee‐ring formation^[^ ^36^, ^37^, ^38^, ^39^ ^]^) and functionality of the resultant patterns.

We exfoliate bulk *h*‐BN crystals (≈1 μm starting lateral dimensions) via ultrasonic‐assisted liquid‐phase exfoliation (UALPE) in a bath ultrasonicator for 12 hours with the PU precursor and 2‐BE mixture; Figure 1a. The resultant dispersion undergoes a centrifugation step at 4000 rpm for 30 min to sediment larger, unexfoliated crystals, allowing extraction of a stable supernatant (top 75%) enriched with few‐layer *h*‐BN flakes. Our approach is a simple yet efficient way to exfoliate 2D *h*‐BN flakes directly with the binder precursor, which is examined by the following set of characterizations.

We first measure the concentration of the *h*‐BN flakes via UV–vis spectroscopy. For this, the dispersion is diluted by 100 times to minimize the scattering losses. The corresponding UV–vis spectrum is shown in Figure 1c, with photograph inset of the diluted *h*‐BN dispersion. Following Beer–Lambert Law and considering negligible scattering losses, and with an absorption (extinction) coefficient of α = 2367 mL mg^−1^ m^−1^ at 300 nm,^[^ ^29^ ^]^ we calculate the *h*‐BN concentration to be ≈6.4 mg mL^−1^. This estimation is confirmed by thermogravimetric analysis (TGA) where we compare the weight loss in the PU precursor+*h*‐BN with pure PU precursor against gradual temperature change; Figure 1d. The lines representing weight percentage of the two systems show a similar trend, but with a 0.6 wt% difference, confirming the amount of *h*‐BN present in the PU precursor + *h*‐BN sample.

Atomic force microscopy (AFM) is next used to statistically estimate the lateral dimension and thickness of the exfoliated *h*‐BN flakes. The average lateral dimension of the exfoliated *h*‐BN flakes is determined to be ≈196.5 nm with a peak at ≈100 nm; Figure 1e. The average thickness of the flakes is significantly reduced to ≈14.8 nm with a peak at ≈7 nm after UALPE when compared to that of the bulk *h*‐BN (≈1 μm) powder; Figure 1f. The layer number of the exfoliated *h*‐BN is hence estimated ≈17–40 according to the measured thickness.

We also acquire Raman spectra of the samples deposited on to Si/SiO_2_ substrates (see methods for details); Figure 1g. Typical measured spectrum from bulk *h*‐BN crystals show a characteristic Raman peak from the E_2*g*_ phonon mode ≈1365.9 cm^−1^.^[^ ^40^ ^]^ On the other hand, the spectrum from the exfoliated *h*‐BN flakes in the ink shows the E_2*g*_ peak at ≈1367.2 cm^−1^. The ≈1.3 cm^−1^ right shift of the E_2*g*_ peak indicates exfoliation from bulk *h*‐BN crystal to *h*‐BN nanosheets.^[^ ^40^ ^]^ The broad peaks at ≈2900 and ≈3100 cm^−1^ originate from the PU polymer precursor.^[^ ^41^ ^]^ Correlations between the E_2*g*_ peak position and the number of layers for *h*‐BN flakes have been previously reported in literature.^[^ ^40^ ^]^ A statistical measurement of Raman spectroscopy (Figure S1, Supporting Information) at 30 different points on the deposited sample shows that most of the E_2*g*_ peaks are located at ≈1367.5 cm^−1^, indicating the likely presence of bilayer *h*‐BN flakes.^[^ ^40^ ^]^ However, this does not agree with our AFM measurements (17–40 layers). This statistically significant discrepancy in the Raman measurement is likely due to doping and other environmental perturbations. This also indicates that E_2*g*_ Raman peak position may not be a reliable way to characterize liquid exfoliated *h*‐BN flakes.

### 2.1 Thin Film Characterization

**Figure 2:**
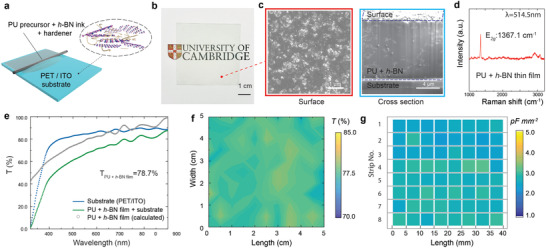
a) A schematic diagram of the K‐bar coating technique for the deposition of PU precursor+*h*‐BN ink. b) Photo of the printed ink layer to show the transparency and c) the corresponding SEM images of the surface morphology and the cross‐sectional view. d) Raman spectroscopy of the coated PU+*h*‐BN thin film showing the presence of exfoliated *h*‐BN flakes. e) Transmittance measurement by UV–vis spectroscopy of the printed thin film. f) Transmittance mapping across a sample area of 5 × 5 cm^2^ and g) capacitance mapping consists of 64 points (8×8 grids) to demonstrate optical and capacitive uniformity of the thin film.

The pre‐polymerized PU is then prepared as a 2‐component resin (i.e., our PU precursor + *h*‐BN ink and hardener) system by mixing the resin and hardener in an optimal ratio of 10:1. We use K‐bar coating (also known as Mayer or Meyer rod coating) to deposit the ink as a thin‐film dielectric for further characterization; **Figure** **2**a. It is a traditional surface coating technique, and uses a wire‐wound rod to deposit liquid ink, usually for small trial investigations.^[^ ^33^ ^]^ In the industry, K‐bar coating is commonly used to test the printability of formulated inks before they are introduced to large‐scale commercial flexographic or gravure printers. This fabrication process is also used for large‐scale un‐patterned coatings in the industry. However, K‐bar coating cannot be directly used to create patterns required for complex electronic circuits. To produce such patterns with the required level of resolution and accuracy while maintaining the material functionality, screen, flexographic, gravure, or inkjet‐printing are more appropriate. This would require optimization of the rheological properties of our formulated ink specific to these printing technologies. The K‐bar coating process requires a typical surface tension < 35–40 mN m^−1^ and a viscosity range of ≈0.01–1 Pa.s.^[^ ^42^ ^]^ The liquid mixture (PU precursor + *h*‐BN ink and hardener) satisfies this requirement and is deposited by K‐bar on a piece of Polyethylene Terephthalate (PET) substrate with a layer of pre‐coated Indium Tin Oxide (ITO) (sheet resistance 350–500 Ω.sq^−1^). The purpose of the ITO coating is to make a conductive bottom contact for subsequent capacitance measurements. A close‐wound K‐bar with 0.64 mm wire diameter is used, giving a wet film thickness of ≈50 μm. A polymerized layer of PU+*h*‐BN thin‐film is slowly formed under room temperature after 48 h. The dry film thickness is estimated to be ≈10 μm.

Figure 2b shows a photograph of the optically transparent 5 × 5 cm^2^ PU+*h*‐BN thin‐film on PET/ITO substrate. The surface morphology and cleaved cross‐section of the dielectric thin‐film are studied by scanning electron microscopy (SEM); Figure 2. The surface morphology image does not show any notable defect that could otherwise be present due to clustering or aggregation of the *h*‐BN flakes, indicating their homogenous distribution within the PU polymer matrix; Figure 2c (left). However, in the cross section image in Figure 2c (right), small “imperfections” are observed. This is very likely due to the small clusters of flaws occurring during the “milling” process by Focused Ion Beam (FIB) we use to cleave the composite. The average thickness of the dielectric is measured by such cross‐section SEM images at different points. Raman spectroscopy is conducted on the PU+*h*‐BN thin‐film, showing the E_2*g*_ peak at ≈1367.1 cm^−1^; Figure 2d.

Adhesion of functional layers to substrates is critical for reliable printed electronics applications. To investigate this, we carry out a pull‐off adhesion test of the coated pure PU and PU+*h*‐BN thin‐films on a glass substrate using a BGD 500 adhesion pull‐off tester. We find that an average force of 1.01 × 10^6^ N m^−2^ (for PU) and 1.07 × 10^6^ N m^−2^ (for PU+*h*‐BN) is required to detach the thin‐film from the glass substrate. This indicates that the incorporation of *h*‐BN flakes and the use of solvent in our processing does not affect the adhesion of the host PU matrix.

The deposited film is next characterized by optical absorption spectroscopy in the 320–900 nm range; Figure 2e. The measured transmittance of the substrate + printed film is 66.5% at 550 nm. Considering the bare substrate optical transmittance (T_*sub*_ of 84.5%), and neglecting reflection at the interfaces and scattering, we estimate that the transmittance of the coated film (T_*PU*+*h*‐*BN film*_) is ≈78.7% at 550 nm. This high optical transparency broadens the scope of potential applications of our dielectric ink, for example, in electroluminescent cells and large area, transparent capacitive sensors.

The deposited thin‐films are also physically uniform and free of pinholes, two critical requirements for dielectric coatings. Unlike the commercially available optically opaque ceramic‐based dielectric inks, which typically require multiple curing and overprints to ensure pinhole‐free construction, this is achieved using a single coating. To investigate the uniformity in T_*PU*+*h*‐*BN film*_, we produce a spatial mapping of a 5 × 5 cm^2^ thin‐film on a 10 × 10 grid (100 points) at 550 nm. Considering an optically uniform substrate, we calculate T_*PU*+*h*‐*BN film*_ of our coated thin‐film and construct a contour plot; Figure 2f. This shows a highly uniform and transparent coating, with an average value of ≈78.0% with a standard deviation of 0.65% at 550 nm. The average root mean square (Rq) surface roughness of the thin film is measured to be 31.1 nm.

We next investigate the thickness uniformity and pin‐hole free construction by fabricating 64 capacitors using an identical sample. For ease of device fabrication and measurement, we first cut this sample into 8 identical strips. On each of these strips, we fabricate eight parallel‐plate capacitors, each with an area of ≈0.5 × 0.5 mm^2^. This gives us a total of 64 capacitors, all fabricated from a single sheet of the thin‐film dielectric. The ITO on the substrate acts as the bottom electrode for these capacitors, while the top electrode is fabricated by evaporating Au/Pt. Capacitance mapping of these 64 samples as they appear in the respective strips is shown in a grid in Figure 2g. We measure an average capacitance of ≈2.72 pF mm^−2^ at 100 kHz, with a standard deviation ≈0.21 pF mm^−2^, representing an <8% variation. This variation could be due to the difference in thickness across the thin‐film. Indeed, we observe a thickness variation of ≈6% from ten different points measured from the cross‐section SEM images. This variation is likely due to the manual coating process we employ (as opposed to an automated K‐bar coater), where a pressure difference could be inadvertently introduced. Nevertheless, we consider the above variation in capacitance between samples within an acceptable range when compared to industrially scalable printing processes.^[^ ^43^ ^]^ We note that, none of the 64 capacitors that we fabricate from this single sample are shorted, indicating pinhole‐free construction of the dielectric from a single coating.

### 2.2 Dielectric Measurements

**Figure 3:**
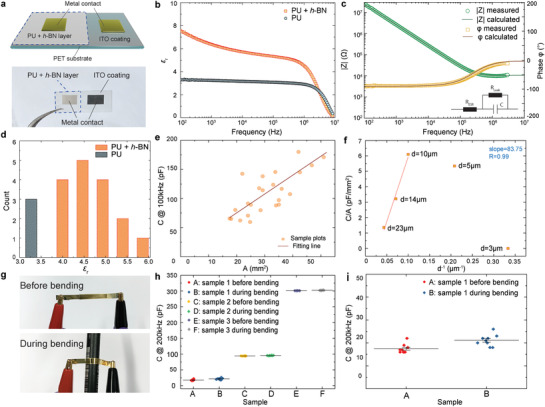
a) Schematic illustration and photograph of the fabricated capacitor with metal contacts for dielectric measurement. b) Comparison of the dielectric constant ε_*r*_ with and without *h*‐BN in the 100 Hz to 10 MHz frequency range. c) Impedance amplitude and phase angle with respect to frequency change 100 Hz to 10 MHz, with an inset of the equivalent circuit. d) Statistics of the ε_*r*_ with and without the incorporation of *h*‐BN. e) Capacitance vs area plot for *h*‐BN incorporated dielectrics. f) Capacitance/area change with respect to thin film thickness. g) Photographs of a capacitor before and during bending for flexibility test. h) Capacitance of three representative samples before and during bending. i) A zoomed‐in capacitance plot of sample 1 in (h).

To further investigate the dielectric properties and their enhancements due to *h*‐BN incorporation in PU, we next use the same K‐bar coating method to fabricate a large number of individual capacitors with different sizes for a series of dielectric measurements. A schematic and a photograph of a typical parallel‐plate capacitor structure to measure the ε_*r*_ value for this experiment are shown in **Figure** **3**a. Parallel‐plate capacitive measurement is one of the most common approaches to evaluate the dielectric properties in a laboratory environment and is based on the simple relationship:

(1) $$C=\frac{\varepsilon_{0}\varepsilon_{r}A}{d}$$

where *C* is the capacitance neglecting fringe effect, ε_0_ is the permittivity of free space, ε_*r*_ is the relative permittivity (dielectric constant) of the dielectric layer between the conductive electrodes, *A* is the area of the capacitor and *d* is the thickness of the dielectric. For our capacitors, *d* (≈10 μm) is estimated from cross‐sectional SEM images. A direct sweep of capacitance is then conducted with respect to a wide frequency range, from 100 Hz to 10 MHz.

The calculated ε_*r*_ from the measurements in Figure 3b reveals a significant enhancement when *h*‐BN is incorporated into the pure PU polymer. At low frequencies (≈100 Hz) more than two‐fold increase in ε_*r*_ is observed; from ε_*r*_ ≈ 3.30 for pure PU to 7.57 for PU+*h*‐BN. A general decrease in ε_*r*_ is observed for both PU and PU+*h*‐BN with an increase in frequency, reaching ε_*r*_ values of 3.01 and 5.10, respectively, at ≈10^6^ Hz, before a steep decline. In particular, for PU+*h*‐BN, the observed reduction is significant. We propose that the very high ε_*r*_ value in the low‐frequency region has a notable contribution from interfacial polarization phenomenon (also known as Maxwell–Wagner–Sillars polarization^[^ ^44^ ^]^); frequently observed in polymer nano‐dielectric systems.^[^ ^4^, ^45^, ^46^ ^]^ This shows a frequency‐dependent contribution to the dielectric response, typically due to the build‐up of space charges at the interface.^[^ ^4^, ^44^ ^]^ These space charges originate from the permittivity and conductivity contrast between the nano‐filler material (*h*‐BN) and the polymer matrix (PU).^[^ ^45^ ^]^ Under an applied electric field in the low‐frequency regime, the space charges tend to accumulate at the vicinity of the two poles of the nanoparticle and are able to respond quickly.^[^ ^47^ ^]^ As the frequency increases (10^3^ to 10^6^ Hz), the contribution from interfacial polarization decreases, giving rise to an overall decrease in the dielectric constant values.^[^ ^4^, ^28^, ^48^ ^]^ The interfacial polarization effect is considered negligible beyond 10^6^ Hz. For our sample, we achieve ε_*r*_ ≈ 4.68 for PU+*h*‐BN and ≈2.92 for plain PU, a 1.6 times increase in ε_*r*_ due to *h*‐BN incorporation at 10^6^ Hz. For frequencies exceeding 10^6^ Hz, the ε_*r*_ value for both PU and PU+*h*‐BN capacitors fall off,^[^ ^19^ ^]^ and eventually diminishes at ≈10^7^ Hz.

The measured impedance (amplitude) |Z| and phase angle between the current and voltage ϕ for the *h*‐BN enhanced PU capacitor with respect to frequency change is also presented in Figure 3c. These reveal a typical R–C equivalent circuit behavior for a parallel‐plate capacitor, following the relationship as:

(2) $$Z=Z_{Re}+jZ_{Im}=R_{ESR}-\frac{j}{\omega C}$$

where *j* is the imaginary number. The equivalent series resistance *R*_*ESR*_ primarily represents the contact resistance from the interface of the electrodes. The leakage current through the dielectric is typically represented by a leakage resistance, *R*_*Leak*_ in parallel to the capacitor in circuit models, as shown in the inset of Figure 3c. In our model, *R*_*Leak*_ is neglected due to the very small measured leakage current of ≈0.2 nA under 10 V DC, giving a high *R*_*Leak*_ of ≈500 GΩ. From the above equation, the impedance value and phase angle can be expressed as:

(3) $$|Z|=\sqrt{R_{ESR}^{2}+{\left(\omega C\right)}^{-2}}$$

and

(4) $$\phi =tan^{-1}(1/\omega R_{ESR}C)$$

Figure 3c shows the phase angle approaches 0° at very high frequencies, leaving only the resistive component in the overall impedance as expected *R*_*ESR*_ (=*Z*_*Re*_). At lower frequency, the phase angle is close to −90°, revealing a dominant capacitive behaviour. The measurement enables the extraction of *R*_*ESR*_ of ≈6.5 kΩ and *C* of ≈ 145 pF. Hence, the corresponding calculated plots (solid lines) for |Z| and ϕ match very well with the measurement; Figure 3c. Due to the experimental limitation, the dielectric loss of our thin‐films is not measured. We next investigate the breakdown voltage of the pure PU and PU+*h*‐BN dielectrics. For this, we apply a high voltage (of up to 1100 V, limited by our setup) on the printed thin‐film (≈10 μm thickness and 25 mm^2^ area) of pure PU and PU+*h*‐BN. The normalised leakage current (mA m^−2^) with respect to the applied electric field is plotted in Figure S2, Supporting Information. It shows that although both the pure PU and PU+h‐BN do not breakdown at 1100 V, the former approaches an onset of breakdown during our measurements in the form of increased leakage currents (from 600 V). This indicates that the dielectric strength of PU+*h*‐BN exceeds 1.1 MV cm^−1^ and the incorporation of *h*‐BN does not compromise the breakdown strength of PU.

To confirm the enhancement of ε_*r*_ by adding *h*‐BN in PU polymer, we fabricate a set of capacitors with and without the incorporation of *h*‐BN. These samples have the same thickness but different sizes, with area varying between 17.5 mm^2^ to 51 mm^2^. The statistical plot of a collection of ε_*r*_ values (neglecting the fringe effect) of these samples is demonstrated in Figure 3d. We take the ε_*r*_ values at 100 kHz, observing that PU+*h*‐BN capacitors have ε_*r*_ values in the range ≈4–6. This is compared with pure PU capacitor having ε_*r*_ values ≈3–3.5. The variation in the ε_*r*_ is likely due to our limited control over the interface between sputter‐coated top electrode and dielectric nanocomposite surface. This could also be due to the variations in the thin‐film interface with the PET/ITO substrate. For the *h*‐BN enhanced capacitors with a thickness of 10 μm, and area ranging from 17.5 to 51 mm^2^, a plot of capacitance, *C* against the area, *A* shows a linear relationship between *C* and *A* as expected; Figure 3e. The relationship between the capacitance per unit area (CA^−1^) and the thickness of the PU+*h*‐BN thin‐film is also investigated. Using the same PU+*h*‐BN dielectric ink, different thickness of the thin‐film is deposited (3, 5, 10, 14, and 23 μm), as shown in Figure 3f. Equation (1) shows that CA^−1^ is proportional to d^−1^. This is matched by the thickness 10, 14, and 23 μm. However, the lower thickness (3 μm, 5 μm) does not indicate a linear relationship and has relatively small capacitance values. This is likely due to non‐uniformity and even discontinuity in these two samples, arising from the de‐wetting of the ink onto the ITO surface after K‐bar coating. This limits the application scope of our dielectric composite to the cases requiring >10 μm thin‐films.

We then carry out a simple bending test to verify the mechanical flexibility of the PU+*h*‐BN dielectric thin‐film. For this, the sample is fabricated by K‐bar coating PU+*h*‐BN ink on PET substrate. The electrodes are thermally evaporated Au (see Figure S3, Supporting Information, for details). An LCR meter (Peak LCR45) is used to measure the capacitance value of the sample at 200 kHz frequency before and during bending, shown in Figure 3g. The bending object is a pen with a bending radius of ≈5 mm. Figure 3h demonstrates 3 samples with distinct capacitance values (sample 1:18 pF, sample 2:94 pF and sample 3:301 pF, respectively) before and during ten times of bending around the pen. A zoomed‐in plot of sample 1 in Figure 3i shows that there is no significant change in the capacitance values, highlighting the mechanical robustness and flexibility of the dielectric thin‐film.

**Figure 4:**
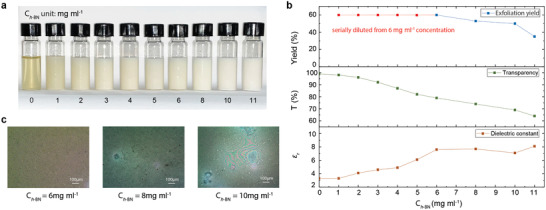
a) Photographs of a set of PU+*h*‐BN dielectric inks with different *h*‐BN concentration (0–11 mg mL^−1^). b) The exfoliation yield, optical transmission T and ε_*r*_ change with respect to *h*‐BN concentration (0–11 mg mL^−1^). c) Optical micrographs of deposited PU+*h*‐BN thin film with three different *h*‐BN concentrations (6, 8, 10 mg mL^−1^).

We also study the *h*‐BN exfoliation yield using different starting material amount, and the role of concentration of the incorporated *h*‐BN on optical transmission, ε_*r*_, and microstructural uniformity of the fabricated dielectric thin‐film. For the exfoliation yield, we investigate a set of concentrations in the dispersions, ranging from 0 to 11 mg mL^−1^; photograph in **Figure** **4**a. The exfoliation yield (%) is calculated by: (the resultant concentration in the dispersion)/(starting concentration of unexfoliated *h*‐BN flakes); see Figure 4b (top). For the resultant *h*‐BN concentration below 6 mg mL^−1^, we use our stock ink (6 mg mL^−1^) to serially dilute into 1–5 mg mL^−1^ concentration samples. Hence, the yield for this range is plotted as 60% (noted in red color in the plot). However, the yield decreases for samples prepared separately with higher starting *h*‐BN concentrations. This suggests that to further increase the resultant *h*‐BN concentration in the dielectric ink, a higher starting concentration is required.

The average optical transmission T_*PU*+*h*−*BN film*_ (at 550 nm) of the thin‐film dielectric shows a gradual reduction in transparency with increasing *h*‐BN concentration; Figure 4b (middle). For 6 mg mL^−1^ concentration, this drops to ≈78%, still acceptable for transparent electronic applications. The ε_*r*_ values of the fabricated thin‐films are enhanced with rising *h*‐BN concentration (Figure 4b (bottom)), and saturates at 6 mg mL^−1^. In general, the effect of filler material concentration on the resultant ε_*r*_ of a polymer nanocomposite can be correlated using the Maxwell Garnett mixing rule.^[^ ^44^ ^]^ This could allow prediction of the effective ε_*r*_ after the incorporation of exfoliated *h*‐BN flakes in the PU host:

(5) $$\varepsilon_{eff}=\varepsilon_{m}\left[1+\frac{\varepsilon_{f}(\varepsilon_{f}-\varepsilon_{m})}{A(1-\phi )(\varepsilon_{f}-\varepsilon_{m})+\varepsilon_{m}}\right]$$

where ϕ is the volume fraction of the filler, ε_*eff*_ and ε_*m*_ and ε_*f*_ are the effective dielectric constant, dielectric constant of matrix, and filler material, respectively, and *A* is the depolarisation factor which relates to the deviation from sphericity (i.e., A = 1/3 for spherical shape). The ϕ value of our dielectric ink (with 6 mg mL^−1^ *h*‐BN) is calculated to be ≈0.7 vol% in the deposited thin‐film. However, the term ε_*f*_ – ε_*m*_ would vanish due to the fact that the ε_*r*_ values for *h*‐BN (≈3.3–3.8^[^ ^50^ ^]^) and PU (≈3–4^[^ ^16^ ^]^) are very close. Therefore the above mixing rule struggles to correlate with our experimental results. Although the distribution in geometry of the exfoliated *h*‐BN could be an additional contributing factor, the reasons behind this disagreement require further systematic investigation and are beyond the scope of our work here. Figure 4c shows three representative optical images of the deposited thin‐film, corresponding to three different *h*‐BN concentrations (6 mg mL^−1^, 8 mg mL^−1^ and 10 mg mL^−1^). The dielectric with 6 mg mL^−1^ *h*‐BN concentration yields uniform and pin‐hole free thin‐film. On the other hand, larger defects and, isolated and agglomerated particles can be clearly seen in the thin‐films with higher *h*‐BN concentrations. Therefore, the optimised concentration of *h*‐BN in the dispersion for the fabrication of an optically transparent dielectric film is indeed 6 mg mL^−1^ *h*‐BN, that is, ≈0.7 vol% in the deposited thin‐film dielectric nanocomposite.

### 2.3 Application in RC Filter

The versatility of our formulated PU+*h*‐BN ink allows for the fabrication of simple electronic circuits, such as low pass filters.

**Figure 5:**
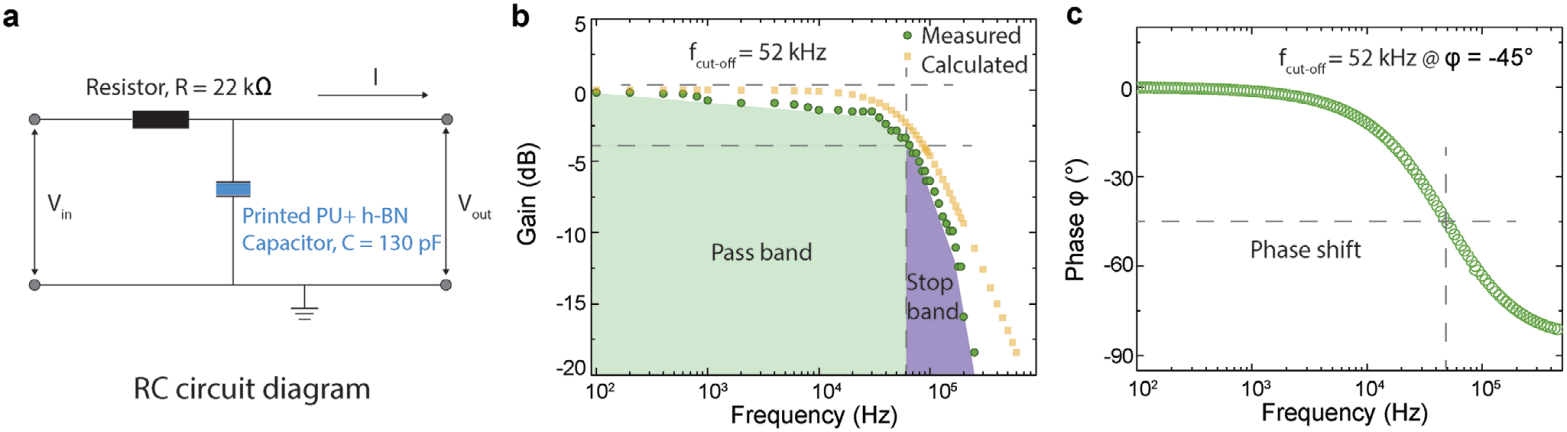
a) Schematic of the RC low pass filter circuit. b) Bode plot of the measured signal gain and c) phase shift against frequency change.

A low pass filter allows low‐frequency signals and blocks or impedes high‐frequency signals. In its simplest form, this circuit can be constructed using a resistor and capacitor, as shown in **Figure** **5**a. A 130 pF PU+*h*‐BN capacitor on PET substrate is used, with a 22 kΩ resistor in series. When operating the RC circuit, an input voltage V_*in*_ with a 10 V amplitude is applied, and its frequency varied from 100 Hz to 250 kHz. The corresponding output voltage V_*out*_ across the capacitor is then measured using an oscilloscope during the frequency sweep. The gain is calculated from the input and output voltage measurements using the following equation, and can be plotted against frequency; Figure 5b.

(6) $$Gain=20log\frac{V_{out}}{V_{in}}$$

The cut‐off frequency *f*_*c*_ (also known as the −3 dB point) is estimated using the equation:

(7) $$f_{c}=\frac{1}{2\pi RC}$$

where *R* = 22 kΩ and *C* = 130 pF. This gives a value of ≈55 kHz, close to the measured *f*_*c*_ value of 52 kHz (Figure 4b; dotted line). In a first‐order RC low pass filter, the slope of gain decrease in the above Bode plot is expected to be −20 dB per decade. This is also closely matched with our measurements.

In Figure 4c, the phase shift (ϕ) is plotted against frequency sweep, calculated by the following equation.

(8) $$\phi =-tan^{-1}(2\pi fRC)$$

It shows phase angle ϕ = –45° at cut‐off frequency 52 kHz as expected for a first‐order RC low pass filter circuit.

### 2.4 Conclusion

Our work demonstrates *h*‐BN enhanced transparent and flexible PU polymer dielectric for the potential applications in printable electronics. We achieve a simple ink formulation by direct UALPE of *h*‐BN in polymer binder and solvent system. With this ink, we demonstrate the fabrication of single‐coat 10‐μm thick, pin‐hole free, flexible dielectric films with high optical uniformity and transparency. We achieve a two‐fold enhancement of ε_*r*_ after the incorporation of *h*‐BN of ε_*r*_ ≈ 7.57 at low frequencies, and up to 1.6 times at 10^6^ Hz. We note that incorporation of *h*‐BN does not compromise the substrate adhesion, and dielectric strength of PU polymer matrix. We also found that thinner films pose a challenge in continuity and therefore limits the application scope to >10 μm dielectric thickness. We then demonstrate the application by the fabrication of a first‐order low pass filter using a PU+*h*‐BN based printed capacitor. Our *h*‐BN enhanced transparent and flexible PU dielctric could be attractive for cost‐effective, large area printable (mm‐to cm‐scale), and flexible applications such as simple electronic circuits, capacitive touch surfaces, and electroluminescent cells. In spite of the challenges in forming ultrathin coatings from such particle‐based ink systems, we envisage that further development in our general approach and rheology of ink would allow for the fabrication of *h*‐BN enhanced, thinner dielectrics suitable for thin‐film‐transistors using conventional printing techniques.

## 3 Experimental Section

##### Ink Formulation

A 10 mg mL^−1^ mixture was made by adding *h*‐BN powder (particle size ≈1 μm, purchased from Sigma Aldrich) into liquid PU polymer and 2‐Butoxyethanol (from Sigma Aldrich) solvent following the recipe (*h*‐BN 1 wt%, PU 41.6 wt%, BC 42 wt%, Hardener 15.4 wt%) under ambient condition. This solution was subjected to 12 h of ultrasonication to achieve the UALPE of *h*‐BN from the bulk. This was followed by centrifuge at 4000 rpm for 30 min. Scanning electron microscopy (SEM) was employed to image the morphology of the sample surface. A thin layer of metal (Au/Pt) was sputter‐coated onto the sample surface to obtain clear images without charging. To observe the cross‐section, a Focused Ion Beam (FIB) instrument (gallium ion beam) attached on a conventional SEM was utilized to directly “mill” the sample perpendicular to the surface via the sputtering process. Thermogravimetric analysis (TGA, QSeries Q50‐1396) was conducted to measure the amount of *h*‐BN in the formulated polymer ink in Argon gas environment, from room temperature to 1000 °C, at a heating rate of 10 °C min^−1^. Tapping mode atomic force microscopy (AFM, Bruker Dimension) was carried out on a drop‐cast sample on Si/SiO_2_ substrate. Raman spectroscopy (Renishaw InVia micro‐Raman spectrometer) was carried out on a similar drop‐cast sample. The excitation wavelength used was 514 nm. The incident power was set below 1 mW to avoid possible thermal damage. The system had a spectral resolution of 1.5 cm^−1^. Optical absorption of *h*‐BN dispersion and transmittance of coated PU+*h*‐BN film were both measured by Cary 7000 UV–vis‐NIR Spectrometer, under absorption and transmission mode, respectively.

##### Thin Film Characterization

For the calculation of the transmittance value T(%) of the PU+*h*‐BN thin film T_*PU*+*h‐BN film*_: the simple relation was used with measured transmission T_*PU+h‐BN film*+*sub*_ of the dielectric and the substrate together, and the measured transmission T_*sub*_ of the bare substrate was used to estimate the transmission spectrum T_*PU*+*h‐BN film*+*sub*_ = T_*sub*_ × T_*PU*+*h‐BN*_. Here, the scattering that might occur within the composite due to the presence of the *h*‐BN flakes and the reflection at the interfaces between air/substrate, substrate/composite, and air/composite were neglected.

A Bruker DektakXT Stylus Profilometer was used to determine the surface roughness (average root mean square Rq) of the deposited thin film. Ten different scanning lines were randomly taken across the thin film surface, acquiring ten different values of Rq. An average value was then calculated.

##### Dielectric Measurement

The parallel‐plate measurements were carried out with a metal‐dielectric‐metal structure. ITO‐coated PET film (thickness 0.2 mm) from Thorlabs was used as the substrate. One pass of the polymer ink was K‐bar coated onto the substrate. This was left for drying for 48 h under ambient conditions. An area having half with deposited film and half without deposition was selected and cut. For capacitor shown in Figure 3b, the total area was around 2–3 cm^2^. Two metal contacts were deposited on the area with and without deposition, respectively, by masking out the areas for deposition. This was done by using depositing Au/Pt, with a contact resistance of ≈10–20 Ω. A four‐probe Cascade Microtech probe station was used with an impedance analyser (Agilent 4294A Precision Impedance Analyser) for the measurements. Four probes were landed onto the two metal contacts (two for each contact). This was to eliminate the contact resistance during measurement. The capacitance, impedance |Z|, phase angle (difference) were measured under a frequency range of 100 Hz to 10 MHz. A set of different concentrations of *h*‐BN in dielectric ink was obtained. The lower *h*‐BN concentration inks (1–5 mg mL^−1^) were achieved by diluting the 6 mg mL^−1^ PU+*h*‐BN stock inks. The relatively higher *h*‐BN concentration inks were obtained by increasing the starting *h*‐BN concentration (before centrifuge). A starting *h*‐BN concentration of 15, 20, and 30 mg mL^−1^ was used to obtain 8, 10, and 11 mg mL^−1^ concentration, respectively.

##### Application

A digital storage oscilloscope (Tektronix TBS2104 Digital Storage Oscilloscope) was used to measure and record the input and output voltage waveform at specific frequencies.

## Conflict of Interest

The authors declare no conflict of interest.

## Author Contributions

X.Z., L.N. designed the experiments. X.Z., L.N., T.C.W., N.M. performed the experiments. X.Z., L.N., G.H., T.C.W., T.H. analysed the data. X.Z., L.N., G.H., D.U. prepared the figures. X.Z., T.H. wrote the manuscript. All authors discussed results from the experiments and commented on the manuscript.

## Supporting information

Supporting Information
