# Supplementary material for: Hexagonal Boron Nitride–Enhanced Optically Transparent Polymer Dielectric Inks for Printable Electronics
Source: Adv Funct Mater. 2020 Jun 2;30(31):2002339. doi: 10.1002/adfm.202002339 (PMC7405982; doi:10.1002/adfm.202002339)
Supplement: Supplementary file 1 — Supporting Information [file ADFM-30-2002339-s001.pdf]

Copyright WILEY-VCH Verlag GmbH & Co. KGaA, 69469 Weinheim, Germany, 2020.

ADVANCED FUNCTIONAL MATERIALS

Supporting Information

for *Adv. Funct. Mater.,* DOI: 10.1002/adfm.202002339

Hexagonal Boron Nitride–Enhanced Optically Transparent Polymer Dielectric Inks for Printable Electronics

*Xiaoxi Zhu, Leonard W. T. Ng, Guohua Hu, Tien-Chun Wu, Doo-Seung Um, Nasiruddin Macadam, and Tawfique Hasan**

Supplementary Information: Hexagonal boron nitride enhanced optically transparent polymer dielectric inks for printable electronics

Xiaoxi Zhu[1], Leonard W. T. Ng[1], Guohua Hu[1,2], Tien-Chun Wu[1], Doo-Seung Um[1], Nasiruddin Macadam[1], Tawfique Hasan[1,*]

[1]Cambridge Graphene Centre, University of Cambridge, Cambridge CB3 0FA, UK.
[2]Department of Electronic Engineering, The Chinese University of Hong Kong, Shatin, Hong Kong S. A. R.
[*]Correspondence to: th270@cam.ac.uk

1 Raman statistics

In order to investigate the correlation between the number of layers of the exfoliated *h*-BN flakes and the E_{2g} peak positions, we conducted statistical Raman analysis. 30 different points are taken randomly across the deposited PU+*h*-BN sample. The Raman spectroscopy measurement is carried out at wavelength 514nm. Supplementary Fig.1 demonstrates the E_{2g} peak positions at 30 different points, having most of the E_{2g} peak at ~ 1367.5 cm^{-1}.

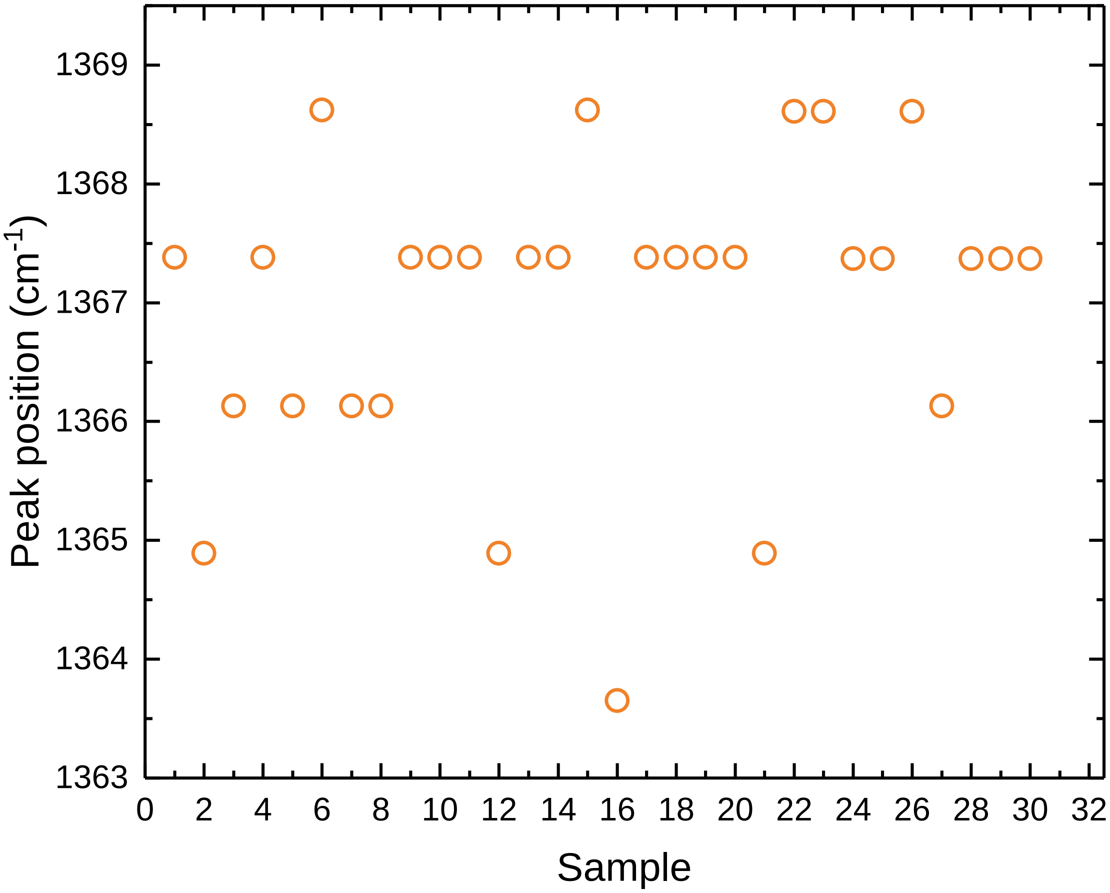

Supplementary Fig. 1 Statistical Raman analysis of the E_{2g} peak positions at 30 different sample points.

2 Breakdown voltage measurement

The breakdown voltage measurement of the pure PU and PU+h-BN dielectrics is shown in Supplementary Fig.2. A high voltage up to 1100 V is applied on the printed thin-film (25 mm^2 area, with a thickness of $\sim$10 μm) of pure PU and PU+h-BN. The current density (mA m^{-2}) with respect to the applied electric field (and the applied voltage) indicates an onset of breakdown for the pure PU at 600 V.

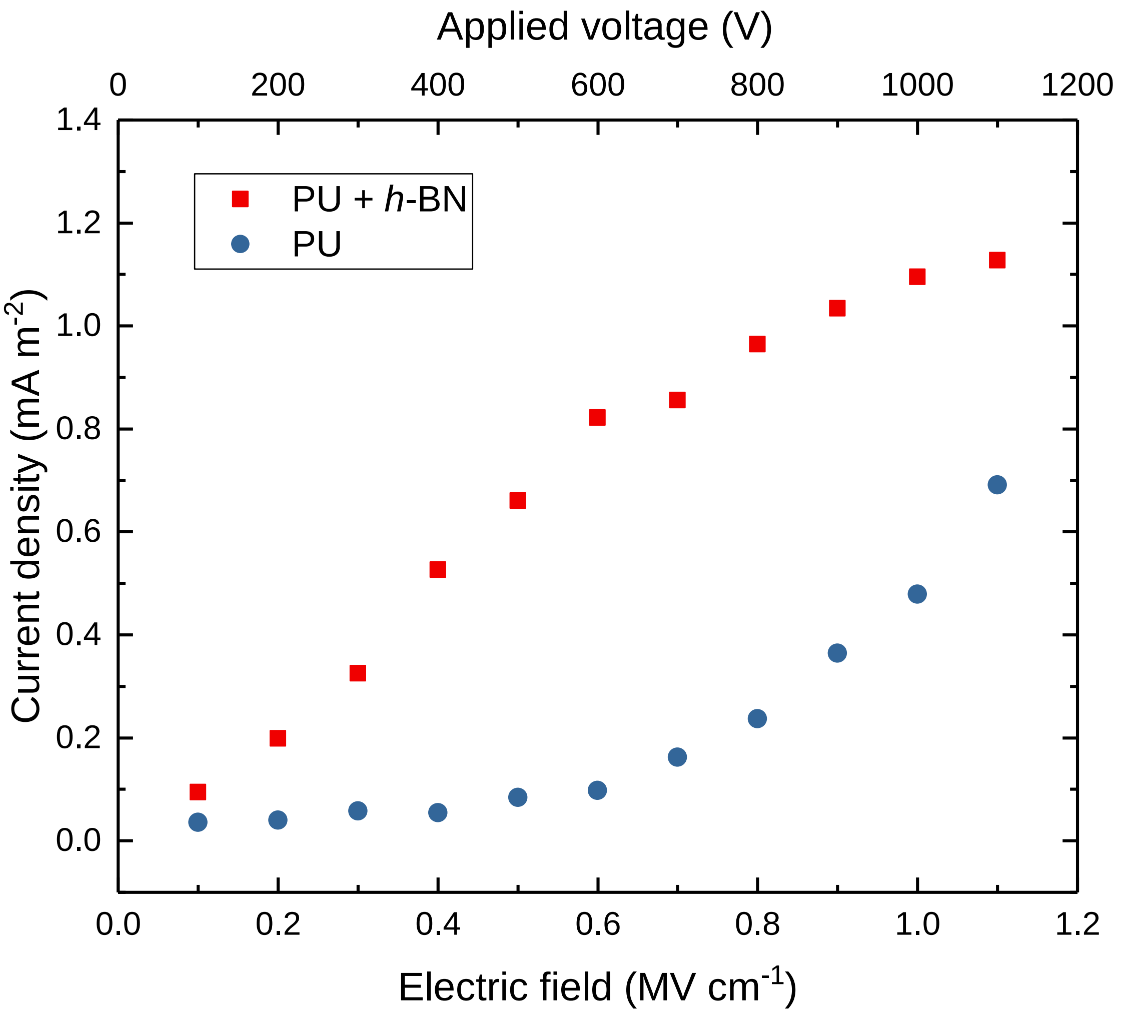

Supplementary Fig. 2 The plot of breakdown voltage measurement for pure PU and PU+h-BN dielectrics.

3 Flexibility test

Supplementary Fig.3 shows the photo of the sample for the flexibility test. It highlights (in dotted blue line) the coated PU+*h*-BN thin film on the PET substrate with a thin layer of thermal evaporated Au ($\sim$50 nm). Another thin layer of Au ($\sim$50 nm) is also deposited above the coated PU+*h*-BN film as the top electrode. The measured area (in dotted red line) is the overlap area of the bottom and top electrode. The cable connections of the LCR meter are introduced at the two ends of the sample stripe, connecting one on the top electrode and the other on the bottom electrode.

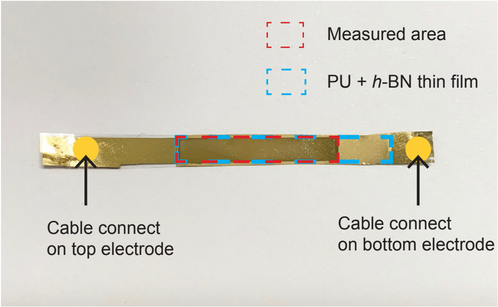

Supplementary Fig. 3 Photo of the PU+*h*-BN thin film sample for flexibility test.
